# The interplay between immunity and aging in *Drosophila*

**DOI:** 10.12688/f1000research.13117.1

**Published:** 2018-02-07

**Authors:** Kathrin Garschall, Thomas Flatt

**Affiliations:** 1 Department of Ecology & Evolution, University of Lausanne, Lausanne, Switzerland; 2 Department of Biology, University of Fribourg, Fribourg, Switzerland

**Keywords:** immunity, immunosenescence, inflammaging, aging, longevity, Drosophila

## Abstract

Here, we provide a brief review of the mechanistic connections between immunity and aging—a fundamental biological relationship that remains poorly understood—by considering two intertwined questions: how does aging affect immunity, and how does immunity affect aging? On the one hand, aging contributes to the deterioration of immune function and predisposes the organism to infections (“immuno-senescence”). On the other hand, excessive activation of the immune system can accelerate degenerative processes, cause inflammation and immunopathology, and thus promote aging (“inflammaging”). Interestingly, several recent lines of evidence support the hypothesis that restrained or curbed immune activity at old age (that is, optimized age-dependent immune homeostasis) might actually improve realized immune function and thereby promote longevity. We focus mainly on insights from *Drosophila*, a powerful genetic model system in which both immunity and aging have been extensively studied, and conclude by outlining several unresolved questions in the field.

## Introduction

The ability to respond to the ubiquitous challenge by pathogens is essential throughout organismal life. The innate immune system provides a phylogenetically conserved strategy against a wide range of pathogens and has been thoroughly studied in—among other organisms—the fruit fly, *Drosophila melanogaster*, a powerful experimental model system.

After intruding microorganisms are recognized through receptors (for example, peptidoglycan recognition proteins, or PGRPs) which bind to common pathogen-associated molecular patterns (PAMPs), such as components of bacterial cell walls, a general immune response is induced ^1^. This includes activation of immune cells, localized production of reactive oxygen species (ROS) and nitrogen oxygen species, and the systemic release of immune effector molecules, so-called antimicrobial peptides (AMPs), which function as humoral “broadband antibiotics” that fight infections ^2^. The expression of AMPs is regulated by the Toll and immune deficiency (IMD) pathways via the activation of the nuclear factor-kappa B (NF-κB) transcription factors *Dif* ( *dorsal-related immunity factor*, Toll pathway) and *relish* (Imd pathway) ^2, 3^. Whereas the Toll pathway is activated by Gram-positive bacteria and fungi, the Imd pathway is predominantly activated by Gram-negative bacteria; however, there is a certain amount of crosstalk between both pathways. The AMPs are produced mainly in the fly’s fat body, an organ that is equivalent to mammalian liver and adipose tissue ^1^. From the fat body, the AMPs are secreted into the hemolymph, the analog of vertebrate blood, where they kill off invading pathogens. The action of the AMPs is supplemented by a cellular defense response mediated by circulating hemocytes ^4, 5^.

Although the immune system contributes vitally to survival and somatic maintenance by preventing mortality and limiting damage imposed by pathogens, it is itself subject to age-dependent deterioration (functional senescence). Moreover, the excessive induction of the immune system, especially at old age, can cause tissue damage and inflammation, which require costly repair ^6^ and thus promote aging. Here, we discuss the interplay between immunity and aging by considering two interrelated questions: how does aging affect immunity, and how does the immune system affect aging and longevity? We do so by examining recent findings in the *Drosophila* model system. We conclude our mini-review by outlining some fundamental but currently unresolved questions in this fascinating field.

## How does aging affect immunity?

The age-dependent decline of immune function and competence, called immuno-senescence, is a general hallmark of the aging process ^7– 9^. In *Drosophila*, a large and growing body of experimental work has established that the immune system is strongly affected by physiological changes that accompany aging and that can lead to a presumably pathological upregulation of antimicrobial defenses and a reduced ability to combat infections ^7, 10– 12^.

### Age-related changes in the microbiome contribute to fly aging

Epithelia exposed to the environment, such as the intestinal epithelium of the digestive tract as well as those of the respiratory and the reproductive systems, serve as major immunological barriers and mediate the interaction between the fly and its microbiome ^1^. Especially the gut, where the control of bacterial growth is essential for effective nutrient uptake, has received much attention in an attempt to understand the physiological effects of its microbiome composition ^13– 15^. The microorganisms residing in the gut and their metabolites strongly shape the transcriptional status of immune genes and can have pervasive effects upon metabolic activity and gut physiology ^16– 19^. During the course of aging, the microbiome changes substantially, especially in terms of increased bacterial diversity and overall abundance ^19– 22^. So, does the microbiome impact fly lifespan? If so, how?

Indeed, several studies have found effects of microbiota on the lifespan of fruit flies. For example, Brummel *et al*., using axenic versus control conditions, showed that the presence of bacteria during the first week of adult life can enhance lifespan but that later in life the presence of bacteria can reduce lifespan. This might suggest a beneficial impact of microbiota early in life but potentially lifespan-shortening effects of the microbiome at old age ^21^. In marked contrast, Ren *et al*., for reasons that remain unclear, failed to find differences in lifespan between flies reared conventionally or flies maintained under reduced bacterial load ^20^. More recently, Clark *et al*. have reported strongly extended lifespan under germ-free axenic conditions ^23^: in discussing the discrepancies among different studies, the authors speculate that, depending on the nutrient environment, the presence of gut-associated microbes might be either beneficial or deleterious. Another possibility is that different host strains exhibit different age-dependent dynamics of their microbiota, thus leading to different lifespan responses when the microbiome is altered ^23^.

In line with important effects of the microbiome on aging, recent studies focusing on microbial communities in the gut have found that age-dependent deregulation of the gut microbiota (dysbiosis) is associated with reduced metabolism, overproliferation of intestinal stem cells (ISCs), and a loss of intestinal barrier integrity, thus contributing to mortality in aging flies ^22, 24– 27^. For instance, Broderick *et al*. showed that the presence of microbiota strongly affects gut morphology and gene expression, inducing a local immune response (mainly through Imd signaling), upregulating the expression of genes for cell differentiation (JAK/STAT pathway), and altering metabolism ^19^. Consistent with these findings, Guo *et al*. observed that the gut epithelia of axenic flies exhibit reduced numbers of mitotically active cells, lower levels of tissue dysplasia, and decreased expression of immune genes ^22^. More recently, Clark *et al*. were able to link the age-dependent shift in microbial composition to gut epithelial deterioration and intestinal barrier failure. The authors showed that changes in the microbiota occur both before and after the loss of gut integrity and that systemic infection—or the mounting of an immune defense response—drives mortality in aged flies ^23^. Thus, even though many details are not yet understood, it seems clear that the age-dependent dynamics of the microbiome can have profound effects on fly aging and physiology.

### Immune and defense response genes are predominantly upregulated with age

Another major, well-documented hallmark of aging in *Drosophila* is the increased expression of genes of the stress and immune responses ^10, 28– 32^. These age-dependent transcriptional changes are shared between the sexes and observed on multiple levels, from pathogen recognition to the increased expression of AMPs produced downstream of both the IMD and the Toll pathways. For instance, Lai *et al*. compared gene expression during male and female aging and saw a strong sex-dependent upregulation with age of genes in the immune system, especially of AMPs ( *Attacins*, *Diptericin*, *Defensin*, *Drosocin*, and *Metchnikowin*) ^33^. Similarly, a recent study by Carlson *et al*. observed that, across several time points, immune effectors and stress-induced genes (for example, *Turandot A* and *C*) are most consistently upregulated across time points from about 3 weeks until 72 days of age ^34^.

Such transcriptional responses to aging seem to be somewhat tissue dependent: in a comparison of multiple male tissues, Zhan *et al*. found that the expression of immune genes in the brain—in marked contrast to abdominal fat tissue exhibiting a strong upregulation of immune expression with age—was downregulated ^35^. In contrast, Kounatidis *et al*. reported a strong *upregulation* of AMPs in the heads and brains of aged flies ^31^, similar to earlier findings ^36^. Chen *et al*. observed that—while the aged fat body upregulates immune gene expression, as has been typically found—IMD activity in the gut is downregulated and that this suppression is a direct response to systemic inflammatory signals ^37^. How such tissue-dependent differences in immune gene expression affect fly aging is not well known, but, overall, most assays of whole flies or of fat body tissue, the main production site of AMPs, suggest that—globally speaking and on average—the upregulation of immune genes with age is the predominant pattern.

What are the causes and the functional consequences of this age-dependent upregulation? On the one hand, the acute and short-term activation of the immune response as a result of injury and pathogen invasion is crucial for survival and induces the repair of affected tissues ^2^. On the other hand, prolonged activation of the immune system at old age can induce chronic inflammation (“inflammaging” ^38^), which is associated with increased host mortality ^23, 31^.

A key factor that likely contributes to a stronger immune activation with age is the increase in microbial load and pathogen diversity during aging ^20– 23, 39^. In support of this, flies reared under strongly reduced microbial load show a less-pronounced age-related increase in the expression of immune genes, yet even under axenic conditions signals of inflammation are observed ^20, 22, 40^. This state of “sterile inflammation” has been attributed in part to the accumulation of senescent cells that secrete a range of inflammatory cytokines, chemokines, and proteases when entering terminal growth arrest in response to stress and DNA damage ^41, 42^. In vertebrates, cellular senescence and the inflammatory phenotype (the “senescence-associated secretory phenotype”, or SASP) are thought to be major drivers of chronic inflammation ^43, 44^. A recent study by Nakamura *et al*. shows that this also occurs in invertebrates: in *Drosophila*, mitochondrial dysfunction in combination with the expression of the oncogene *Ras* induces both cellular senescence (accompanied by activation of JNK signaling) and systemic expression of proinflammatory cytokines of the *unpaired* (upd) family that impact tissue homeostasis ^45^. In a similar vein, Chen *et al*. observed that, during aging, cellular senescence in the fat body leads to a systemic inflammatory response which deregulates IMD signaling in the midgut. They further showed that the observed deregulation of immune gene expression is mainly driven by the age-related decline in nuclear Lamin-B, a marker for cellular senescence ^46^, which leads to a loss of heterochromatin-mediated repression ^37^.

In addition to immune activation by microbial imbalance, chronic inflammation in the gut is induced in part by overproliferation of ISCs ^22, 24, 26, 47^. For example, Li *et al*. propose that the upregulation of JAK/STAT signaling through inflammatory signals (for example, *upd2* and *upd3*) initiates the loss of tissue homeostasis, even in the absence of a microbiome ^27^. The authors suggest that the age-dependent increase in JAK/STAT signaling causes the loss of gut compartmentalization, thereby facilitating a pathological shift in microbiota composition, which further induces the local immune response ^27^. Thus, overproliferation of ISCs and accumulation of undifferentiated intestinal cells weaken the epithelial barrier so that ingested bacteria can “leak” into the body cavity, causing systemic or chronic infections that lead to mortality ^22, 23, 26^.

Another cause of increased inflammatory signaling during aging might be oxidative stress, a notion supported by transcriptional similarities in patterns of immune gene expression between aging and oxidative stress ^29, 30, 36^. Mitochondria are a major source of cellular ROS, and impaired mitochondrial function has been proposed to strongly contribute to aging ^48^. Damaged mitochondria and deregulation of oxidative phosphorylation cause an inflammatory response ^49^, activate NF-κB signaling ^50^, and can contribute to intestinal dysfunction ^26^. A recent study by Rana *et al*. shows that preventing morphological changes in the mitochondria by overexpression of *Dynamin-related protein 1* ( *Drp1*) improves mitochondrial respiratory function and increases lifespan ^51^, but the authors did not assess immune transcription. Interestingly, mutants of mitochondrial peroxiredoxins ( *dPrx3* and *dPrx5*), involved in the regulation of ROS levels, exhibit increased activation of the immune response, whereas overexpression of these peroxiredoxins extends lifespan and delays the age-related inflammatory response ^52^.

In summary, it is still not entirely clear whether the strong age-dependent upregulation of immune transcription represents an adaptive and necessary physiological response in order to deal with the increase in pathogen load or whether it reflects the age-progressive loss of the ability to fight off microbial infections. The overall consensus in the field seems to be that increased old-age immune expression probably represents a state of immunopathology (that is, inappropriate hyperactivation of the immune system and chronic inflammation). Moreover, since the immune system also plays an important role in the defense against non-biotic stresses and activates tissue repair mechanisms, the state of chronic inflammation that is commonly observed during aging is likely the cumulative outcome of several aspects of physiological deterioration.

### Age-dependent decline of cellular and realized immune responses

What has been learned about age-related changes in cellular and realized immune responses (that is, the actual resistance to infection with different pathogens) in *Drosophila*? The evidence available to date suggests that overall both tend to decline with age in flies, even though some exceptions or discrepancies have been reported as well.

The cellular immune defense of *Drosophila* is performed by three distinct groups of hemocytes that respond to infection with encapsulation (lamellocytes), melanization (crystal cells), or phagocytosis (plasmatocytes) ^2, 53^. Phagocytes initially reduce pathogen load, contribute to an inflammatory state, and play an important role in the clearance of pathogens and in the regenerative response ^54– 56^. For instance, Guillou *et al*. found that mutants of *croquemort* ( *crq*), a scavenger receptor required for phagocytosis in the plasmatocytes, are vulnerable to environmental microbes and exhibit a chronic state of Imd activation, premature gut dysfunction, and reduced lifespan ^57^. With advancing age, both the number and the phagocytic activity of hemocytes decrease ^58, 59^. Interestingly, Horn *et al*. found that the processing of phagocytosed vesicles is impaired with age, indicating a possible link to autophagy in hemocyte aging and providing positive evidence for a less-efficient cellular defense at advanced age ^59^.

In terms of the ability of flies to clear out pathogens as a function of age, the observations available to date are somewhat inconsistent: for example, Ramsden *et al*. found no effect of age on bacterial clearance ^60^, yet other studies have observed that clearance ability differs strongly among genotypes and either declines or even increases with age ^61, 62^. Thus, it is not necessarily always the case that old flies are worse at clearing out infections, as might be expected. Notably, Duneau *et al*. have found that most bacterial infections are in fact not cleared out but rather persist at low levels of pathogen burden ^63^; in turn, this could explain the typically observed upregulation of immune gene expression and symptoms of chronic inflammation. In support of a dysfunctional senescent immune response at old age, Zerofsky *et al*. found that the inducibility of the AMP response declines with age: under systemic infection, young flies reach peak expression of *Diptericin* after 12 hours, whereas old flies exhibit delayed but stronger and prolonged expression. In contrast, however, AMP induction in response to heat-killed bacteria was significantly weaker in older flies. This indicates that the reduced inducibility of the AMP response in old flies causes a major disadvantage in terms of the flies’ ability to curb bacterial growth, thus leading to delayed but stronger expression ^11^.

Together with the fact that the susceptibility to infections increases with age in flies ^60, 64– 66^, the results of Zerofsky *et al*. (above) strongly suggest that the functional capacity of the innate immune system declines with age in *Drosophila*. Interestingly, however, this immunosenescence might not always involve a decline in clearance ability: Ramsden *et al*. found that the ability of flies to clear an *Escherichia coli* infection is unaffected by age but that the ability to survive an infection strongly declines during aging. A further complication in understanding links between immunity and aging is that the senescent deterioration of the immune system might be sex specific: in male flies, susceptibility to the entomopathogenic fungus *Beauveria bassiana* is increased with age because of a failure of the barrier defenses, whereas female flies exhibit systemic immune senescence ^64^. Yet perhaps the most fundamental question is this: what causes the death of infected flies at old age? To date, our understanding of this issue remains very poor; ultimately, host survival will depend on the balance between the defensive response to infection, the consequences of pathogen-inflicted damage, and the effects of self-harm caused by a potentially prolonged, chronic immune response ^67^. We now turn to discussing the other side of the coin, namely how does the immune system impact senescence and organismal lifespan?

## How does immunity affect aging and longevity?

### Costs of immunity and trade-offs

As vital as the immune system is for survival, mounting an immune response is also metabolically costly ^68^ and bears the potential of inflicting autoimmune damage that contributes to aging ^69– 71^. That the immune system is costly is apparent from the tight regulation of its induction and the existence of physiological and evolutionary trade-offs between immunity and other life history traits such as developmental time ^72– 74^, reproduction ^11, 73, 75– 78^, and lifespan ^70, 73, 74, 79, 80^. In terms of detrimental effects of immune induction, Pursall and Rolff showed that, in the mealworm beetle *Tenebrio molitor*, provoking an immune response early in life causes reduced lifespan ^70^. In a more recent study of this beetle system, Khan *et al*. found that the immune-pathological consequences of infection on the Malpighian tubules can be ameliorated by limiting the expression of phenoloxidase (PO), thereby restoring normal lifespan after infection ^81^. Studies in insects have also yielded important insights into the antagonistic relationship between reproduction and immunity, as comprehensively reviewed by Schwenke *et al*. ^77^. For instance, Zerofsky *et al*. observed that activation of the immune system by heat-killed bacteria decreases female fecundity and that this effect depends on the NF-κB transcription factor *relish*. Conversely, mating can suppress the immune system by upregulating the production of a gonadotropic ^78^ but immunosuppressive hormone called juvenile hormone, as recently shown by Schwenke and Lazzaro ^82^. Thus, immunity can induce reproductive costs, and mating can impair immunity in flies. The fat body likely plays a central role in mediating many of the physiological trade-offs that involve immunity, since it represents a major site of both metabolic and endocrine functions important for growth, reproduction, and lifespan (for example, nutrient sensing, storage and utilization, and endocrine signaling) and of immune function (for example, expression of AMPs) ^83^.

### Metabolic consequences of an immune response

An important aspect of trade-offs is that they are often mediated by competitive energy allocation and thus by metabolism. For example, during an immune response, energy metabolism is strongly disrupted and characterized by decreased insulin/insulin-like growth factor signaling (IIS) ^84– 86^, especially in response to systemic activation of Toll signaling ^86^. Conversely, a long-lived mutant of the insulin receptor substrate *chico* shows strongly improved realized immunity and increased AMP induction after bacterial infection ^87^. Becker *et al*. showed that IIS pathway mutants induce AMP expression through the IIS transcription factor FOXO independent of NF-κB activation and that, under conditions of energy shortage or stress, FOXO induces AMPs in several tissues, thereby probably ensuring epithelial defense ^88^.

Other findings also underscore fundamental links between the immune response and metabolism. Mutants for *activating transcription factor 3* ( *Atf3*), for example, show a transcriptional response characteristic of inflammatory stress and starvation while accumulating fat, indicating a loss of homeostasis of metabolism and immunity ^89^. Clark *et al*. identified *Mef2* ( *myocyte enhancer factor 2*) as a switch from anabolism to immune gene expression in response to bacterial infection ^90^. In the healthy fly, phosphorylated *Mef2* regulates the synthesis of fat and glycogen in the fat body and, in response to infection, *Mef2* promotes the expression of several AMPs ^90^. Interestingly, *Mef2* is part of a pathway, the p38 MAP kinase (p38K)/Mef2/MnSOD pathway, that co-regulates stress and lifespan in flies *in vivo* ^91^.

The crosstalk between metabolism and immunity can impose severe costs to mounting a persistent immune response; for example, during infection with *Mycobacterium marinum*, prolonged activation of FOXO can severely deplete nutrient storage and result in lethal “wasting” ^85^. Similarly, Rera *et al*. found that flies overexpressing the AMP *Drosomycin* exhibit a strongly altered metabolic profile, including depletion of glycogen and triglyceride storage, and impaired IIS ^26^. Changes in lipid metabolism have also been associated with an age-related decline in gut function ^47^, and Karpac *et al*. report that such changes are caused by an increase in FOXO and JNK activity in intestinal enterocytes, leading to loss of lipid homeostasis in the gut ^92^.

Beyond these findings, there is compelling evidence that the age-related dysregulation of metabolism, intestinal dysfunction, and the chronic upregulation of the immune system are tightly linked ^15, 23, 26, 27^, yet the chronological order and the mechanistic basis of the effects that lead to the loss of metabolic and immune homeostasis remain poorly understood. Thus, all in all, there are very good reasons to think that metabolism, immunity, and aging are linked in fundamental but still little-understood ways.

### Chronic immune activity shortens life but reduced NF-κB signaling extends it

The metabolically costly and potentially autoreactive nature of the innate immune response requires tight regulation in order to ensure effective pathogen control and limited self-harm ^6, 93^. As discussed previously, immune hyperactivation is a generally observed characteristic of aging ^10, 30, 32, 94^. On the other hand, there is growing evidence that the dysregulation of the immune system strongly contributes to aging. Thus, an interesting question in this context is whether and how immune genes impact organismal lifespan.

Overexpression of the Toll receptor in the fly gut decreases lifespan, and two independent studies reported that a loss-of-function mutant of *Dif* outlives wild-type flies ^95, 96^. Perhaps the clearest findings come from studies of chronic activation of IMD signaling: transgenic overexpression of *PGRP-LC* ^8^ and *PGRP-LE* ^39^ in the fly fat body shortens lifespan. Similarly, flies that overexpress *PGRP-LE* in the gut are shorter lived ^39^. Consistent with these results, it has been found that loss-of-function mutations or RNA interference against negative regulators of IMD signaling ^97^, such as *caudal* ^98^, *trabid* and *pirk* ^99^, and *PGRP-SC* and *PGRP-LB* ^100^, dramatically decreases lifespan, demonstrating the existence of detrimental effects upon the lifespan of immune hyperactivity. In line with this interpretation, overexpression of the negative regulator PGRP-SC2 in the gut increases lifespan by contributing to microbial balance and epithelial homeostasis ^22^.

The importance of a well-regulated interaction between the gut microbiome and the intestinal barrier epithelium is highlighted by a study by Li *et al*., who showed that reduced JAK/STAT signaling delays dysbiosis and extends lifespan ^27^. Yet other examples for the negative impact of dysregulated immunity are mutants of *big bang* (BBG), a gene important for the organization of septate junctions between gut endothelial cells ^101^. Bonnay *et al*. found that BBG mutants exhibit chronically activated immunity due to reduced intestinal barrier function and thus suffer from systemic infections. These flies have shorter lives than average, a phenotype that can be rescued by the administration of antibiotics ^101^.

Effects of chronic NF-κB signaling have also been associated with age-related neurodegeneration in the *Drosophila* brain and nervous system ^95, 102^. A mutant for *defense repressor 1* ( *dnr1*), a negative regulator of IMD, exhibits shortened lifespan as well as neuropathology accompanied by increased AMP levels ^103^. Kounatidis *et al*. recently found that the expression of AMPs ( *Drosocin*, *AttacinC*, and *CecropinA1*) increases with age in the fly brain, leading to progressive neurodegeneration and reduced lifespan; suppression of IMD signaling in the glia cells, on the other hand, led to improved locomotion, an altered metabolic profile, and increased lifespan ^31^.

However, AMPs expressed through NF-κB signaling are not necessarily always detrimental: Loch *et al*. have reported that overexpression of *CecropinA1* and *Drosocin* can actually increase lifespan (possibly by helping to prevent bacterial dysbiosis ^104^) yet simultaneously causes reduced lifetime activation of IMD and JAK/STAT signaling. In addition, it has been shown that flies that overexpress *Diptericin* exhibit increased antioxidant defense and increased tolerance to hyperoxia ^105^.

Therefore, the work reviewed here suggests that a prolonged dysregulation of NF-κB signaling is highly detrimental for lifespan and that interventions that decrease immune activity—at least in the absence of pathogens—can potentially improve tissue homeostasis, delay aging, and prolong life, probably by alleviating the detrimental effects of immune hyperactivity and chronic inflammation. Thus, while the adaptive value of a properly functioning immune system is obvious, it is becoming increasingly clear that immunity represents a “double-edged sword”.

### The role of immunity in the evolution of aging

Might there be a connection between the evolution of longer life and the evolution of immune function? Several studies have used laboratory artificial selection experiments to breed flies for postponed aging and increased lifespan; remarkably, despite differences in the methodological details, these studies indicate that evolutionary changes in immunity might make an important contribution to the evolution of longevity. Remolina *et al*. sequenced the genomes of fly populations after 50 generations of selection for increased lifespan and found a statistical over-representation of longevity candidate genes involved in “defense response to fungus” ^106^. Similarly, another “evolve-and-resequence” study, by Carnes *et al*. ^107^, examined the genomic and transcriptomic basis of longevity in an independent long-term selection experiment for postponed aging ^108^. Although the authors did not observe any over-representation of immune genes at the genomic level, flies from the long-lived selected populations had a lower expression of immune transcripts than unselected controls; genes identified as candidates for postponed senescence included *Metchnikowin*, *CecropinA1*, and *CecropinA2* in females and *PRGP-LF* and *CecropinA2* in males ^107^. Given the negative impact of too much NF-κB expression on lifespan (discussed above), the reduced expression of AMPs in long-lived selected flies makes perfect sense, but further experiments will be required to firmly establish causation. In general, still, very little is known about whether and how genes of the immune system affect organismal lifespan.

## Outlook and open questions

We end by singling out three unresolved questions which, in our minds, would be worth studying in future work, yet there are obviously many more that would be interesting to address.

### 1. What are the age-related changes in other immune active organs?

Most previous work in the field has focused on the gut as a central element that shapes the relationship among the fly microbiome, tissue homeostasis, and inflammatory signals ^15, 97, 109^. However, we clearly lack knowledge about age-dependent changes in other organs that play an important role in immunity. For example, the fat body, as a site of both metabolic and immunological activity, is an obviously interesting organ for much closer investigation in this respect, and many other organs would be of interest as well. More generally, tissue-specific immune signatures need to be considered in the context of the systems physiology of the whole fly, and this is clearly an area where more work is needed.

### 2. Does pathogen evolution within the host play a role in the systemic inflammation observed during aging?

In the light of gut physiology and tissue homeostasis, the *Drosophila* microbiome has gained a lot of attention ^15, 16, 19, 110^. The evolutionary biologist Graham Bell has proposed that senescence might be caused by infections that outcompete the host immune response in an evolutionary arms race ^111^. With the rapid advances in sequencing technology and genomics, it would be very interesting to study pathogen evolution over the course of the *Drosophila* lifetime. This would allow us to investigate whether the evolution of commensals or pathogens to avoid and escape the immune system could explain the increased dysregulation of the host immune system observed with age.

### 3. What is the role of the Toll pathway in affecting aging and lifespan?

Whereas the dysregulation of IMD signaling has been shown to be detrimental in multiple studies ^22, 39, 98– 100^, the role of Toll signaling in aging and lifespan has not yet been studied in depth. This might be because of the focus on aging in the gut, as AMP expression in epithelia seems to be regulated predominantly through the IMD pathway ^112^. Conversely, Toll is activated mainly during systemic infection and plays an important role in the fat body to regulate growth and metabolism ^86^ and in activating hemocytes ^113^. Both the contribution to systemic NF-κB activation and the metabolic impact of Toll signaling would merit close investigation with regard to aging and lifespan in the fly.

## Conclusions

The *Drosophila* immune system is strongly affected by the degenerative processes that accompany aging. The widely observed state of chronic inflammation, a loss of cellular immunity, and the gradual deterioration of protective epithelial barriers all contribute to the functional senescence and increased pathogen susceptibility of aging flies ^7, 114^. In addition, the immune system itself can accelerate aging by inflicting collateral tissue damage ^115^ and by impacting metabolism ^84– 86^. Interventions that reduce the age-associated dysregulation of NF-κB signaling have revealed that the immune system can have a strong impact on organismal aging and lifespan ^1, 8, 22, 39, 100^. Great strides have also been made in our understanding of the physiological processes that occur during aging in the *Drosophila* gut ^23, 27^. Despite all of this impressive progress, the mechanistic connections between immunity and aging and longevity remain poorly understood, and there is much exciting work left to be done in this field.

## References

[1] Ferrandon D Imler JL Hetru C: The *Drosophila* systemic immune response: sensing and signalling during bacterial and fungal infections. *Nat Rev Immunol.* 2007;7(11):862–74. 10.1038/nri2194 17948019

[2] Lemaitre B Hoffmann J: The host defense of *Drosophila melanogaster*. *Annu Rev Immunol.* 2007;25:697–743. 10.1146/annurev.immunol.25.022106.141615 17201680

[3] De Gregorio E Spellman PT Tzou P: The Toll and Imd pathways are the major regulators of the immune response in *Drosophila*. *EMBO J.* 2002;21(11):2568–79. 10.1093/emboj/21.11.2568 12032070 PMC126042

[4] Nehme NT Liégeois S Kele B: A model of bacterial intestinal infections in *Drosophila melanogaster*. *PLoS Pathog.* 2007;3(11):e173. 10.1371/journal.ppat.0030173 18039029 PMC2094306

[5] Elrod-Erickson M Mishra S Schneider D: Interactions between the cellular and humoral immune responses in *Drosophila*. *Curr Biol.* 2000;10(13):781–4. 10.1016/S0960-9822(00)00569-8 10898983

[6] Read AF Graham AL Råberg L: Animal defenses against infectious agents: is damage control more important than pathogen control. *PLoS Biol.* 2008;6(12):e4. 10.1371/journal.pbio.1000004 19222305 PMC2605932

[7] Müller L Fülöp T Pawelec G: Immunosenescence in vertebrates and invertebrates. *Immun Ageing.* 2013;10(1):12. 10.1186/1742-4933-10-12 23547999 PMC3637519

[8] DeVeale B Brummel T Seroude L: Immunity and aging: the enemy within? *Aging Cell.* 2004;3(4):195–208. 10.1111/j.1474-9728.2004.00106.x 15268753

[9] Zhang R Chen HZ Liu DP: The Four Layers of Aging. *Cell Syst.* 2015;1(3):180–6. 10.1016/j.cels.2015.09.002 27135911

[10] Pletcher SD Macdonald SJ Marguerie R: Genome-wide transcript profiles in aging and calorically restricted *Drosophila melanogaster*. *Curr Biol.* 2002;12(9):712–23. 10.1016/S0960-9822(02)00808-4 12007414

[11] Zerofsky M Harel E Silverman N: Aging of the innate immune response in *Drosophila melanogaster*. *Aging Cell.* 2005;4(2):103–8. 10.1111/j.1474-9728.2005.00147.x 15771614

[12] Iliadi KG Knight D Boulianne GL: Healthy aging - insights from *Drosophila*. *Front Physiol.* 2012;3:106. 10.3389/fphys.2012.00106 22529821 PMC3328947

[13] Wong AC Luo Y Jing X: The Host as the Driver of the Microbiota in the Gut and External Environment of *Drosophila melanogaster*. *Appl Environ Microbiol.* 2015;81(18):6232–40. 10.1128/AEM.01442-15 26150460 PMC4542222

[14] Wong AC Dobson AJ Douglas AE: Gut microbiota dictates the metabolic response of *Drosophila* to diet. *J Exp Biol.* 2014;217(Pt 11):1894–901. 10.1242/jeb.101725 24577449 PMC4037322

[15] Buchon N Broderick NA Lemaitre B: Gut homeostasis in a microbial world: insights from *Drosophila melanogaster*. *Nat Rev Microbiol.* 2013;11(9):615–26. 10.1038/nrmicro3074 23893105

[16] Dobson AJ Chaston JM Douglas AE: The *Drosophila* transcriptional network is structured by microbiota. *BMC Genomics.* 2016;17(1):975. 10.1186/s12864-016-3307-9 27887564 PMC5124311

[17] Newell PD Douglas AE: Interspecies interactions determine the impact of the gut microbiota on nutrient allocation in *Drosophila melanogaster*. *Appl Environ Microbiol.* 2014;80(2):788–96. 10.1128/AEM.02742-13 24242251 PMC3911109

[18] Erkosar B Defaye A Bozonnet N: *Drosophila* microbiota modulates host metabolic gene expression via IMD/NF-κB signaling. *PLoS One.* 2014;9(4):e94729. 10.1371/journal.pone.0094729 24733183 PMC3986221

[19] Broderick NA Buchon N Lemaitre B: Microbiota-induced changes in *Drosophila melanogaster* host gene expression and gut morphology. *mBio.* 2014;5(3):e01117–14. 10.1128/mBio.01117-14 24865556 PMC4045073

[20] Ren C Webster P Finkel SE: Increased internal and external bacterial load during *Drosophila* aging without life-span trade-off. *Cell Metab.* 2007;6(2):144–52. 10.1016/j.cmet.2007.06.006 17681150

[21] Brummel T Ching A Seroude L: *Drosophila* lifespan enhancement by exogenous bacteria. *Proc Natl Acad Sci U S A.* 2004;101(35):12974–9. 10.1073/pnas.0405207101 15322271 PMC516503

[22] Guo L Karpac J Tran SL: PGRP-SC2 promotes gut immune homeostasis to limit commensal dysbiosis and extend lifespan. *Cell.* 2014;156(1–2):109–22. 10.1016/j.cell.2013.12.018 24439372 PMC3928474

[23] Clark RI Salazar A Yamada R: Distinct Shifts in Microbiota Composition during *Drosophila* Aging Impair Intestinal Function and Drive Mortality. *Cell Rep.* 2015;12(10):1656–67. 10.1016/j.celrep.2015.08.004 26321641 PMC4565751

[24] Biteau B Hochmuth CE Jasper H: JNK activity in somatic stem cells causes loss of tissue homeostasis in the aging *Drosophila* gut. *Cell Stem Cell.* 2008;3(4):442–55. 10.1016/j.stem.2008.07.024 18940735 PMC3225008

[25] Buchon N Broderick NA Poidevin M: *Drosophila* intestinal response to bacterial infection: activation of host defense and stem cell proliferation. *Cell Host Microbe.* 2009;5(2):200–11. 10.1016/j.chom.2009.01.003 19218090

[26] Rera M Clark RI Walker DW: Intestinal barrier dysfunction links metabolic and inflammatory markers of aging to death in *Drosophila*. *Proc Natl Acad Sci U S A.* 2012;109(52):21528–33. 10.1073/pnas.1215849110 23236133 PMC3535647

[27] Li H Qi Y Jasper H: Preventing Age-Related Decline of Gut Compartmentalization Limits Microbiota Dysbiosis and Extends LifeSpan. *Cell Host Microbe.* 2016;19(2):240–53. 10.1016/j.chom.2016.01.008 26867182 PMC5106289

[28] Highfill CA Reeves GA Macdonald SJ: Genetic analysis of variation in lifespan using a multiparental advanced intercross *Drosophila* mapping population. *BMC Genet.* 2016;17:113. 10.1186/s12863-016-0419-9 27485207 PMC4970266

[29] Landis G Shen J Tower J: Gene expression changes in response to aging compared to heat stress, oxidative stress and ionizing radiation in *Drosophila melanogaster*. *Aging (Albany NY).* 2012;4(11):768–89. 10.18632/aging.100499 23211361 PMC3560439

[30] Landis GN Abdueva D Skvortsov D: Similar gene expression patterns characterize aging and oxidative stress in *Drosophila melanogaster*. *Proc Natl Acad Sci U S A.* 2004;101(20):7663–8. 10.1073/pnas.0307605101 15136717 PMC419663

[31] Kounatidis I Chtarbanova S Cao Y: NF-κB Immunity in the Brain Determines Fly Lifespan in Healthy Aging and Age-Related Neurodegeneration. *Cell Rep.* 2017;19(4):836–48. 10.1016/j.celrep.2017.04.007 28445733 PMC5413584

[32] Seroude L Brummel T Kapahi P: Spatio-temporal analysis of gene expression during aging in *Drosophila melanogaster*. *Aging Cell.* 2002;1(1):47–56. 10.1046/j.1474-9728.2002.00007.x 12882353

[33] Lai C Parnell LD Lyman RF: Candidate genes affecting *Drosophila* life span identified by integrating microarray gene expression analysis and QTL mapping. *Mech Ageing Dev.* 2007;128(3):237–49. 10.1016/j.mad.2006.12.003 17196240

[34] Carlson KA Gardner K Pashaj A: Genome-Wide Gene Expression in relation to Age in Large Laboratory Cohorts of *Drosophila melanogaster*. *Genet Res Int.* 2015;2015:835624. 10.1155/2015/835624 26090231 PMC4454753

[35] Zhan M Yamaza H Sun Y: Temporal and spatial transcriptional profiles of aging in *Drosophila melanogaster*. *Genome Res.* 2007;17(8):1236–43. 10.1101/gr.6216607 17623811 PMC1933522

[36] Girardot F Lasbleiz C Monnier V: Specific age related signatures in *Drosophila* body parts transcriptome. *BMC Genomics.* 2006;7:69. 10.1186/1471-2164-7-69 16584578 PMC1481561

[37] Chen H Zheng X Zheng Y: Age-associated loss of lamin-B leads to systemic inflammation and gut hyperplasia. *Cell.* 2014;159(4):829–43. 10.1016/j.cell.2014.10.028 25417159 PMC4243052

[38] Franceschi C Bonafè M Valensin S: Inflamm-aging. An evolutionary perspective on immunosenescence. *Ann N Y Acad Sci.* 2000;908:244–54. 10.1111/j.1749-6632.2000.tb06651.x 10911963

[39] Libert S Chao Y Chu X: Trade-offs between longevity and pathogen resistance in *Drosophila melanogaster* are mediated by NFkappaB signaling. *Aging Cell.* 2006;5(6):533–43. 10.1111/j.1474-9726.2006.00251.x 17129215

[40] Kounatidis I Ligoxygakis P: *Drosophila* as a model system to unravel the layers of innate immunity to infection. *Open Biol.* 2012;2(5):120075. 10.1098/rsob.120075 22724070 PMC3376734

[41] Coppé JP Desprez PY Krtolica A: The senescence-associated secretory phenotype: the dark side of tumor suppression. *Annu Rev Pathol.* 2010;5:99–118. 10.1146/annurev-pathol-121808-102144 20078217 PMC4166495

[42] Pawelec G Goldeck D Derhovanessian E: Inflammation, ageing and chronic disease. *Curr Opin Immunol.* 2014;29:23–8. 10.1016/j.coi.2014.03.007 24762450

[43] Campisi J: Aging, cellular senescence, and cancer. *Annu Rev Physiol.* 2013;75:685–705. 10.1146/annurev-physiol-030212-183653 23140366 PMC4166529

[44] Neves J Demaria M Campisi J: Of flies, mice, and men: evolutionarily conserved tissue damage responses and aging. *Dev Cell.* 2015;32(1):9–18. 10.1016/j.devcel.2014.11.028 25584795 PMC4450349

[45] Nakamura M Ohsawa S Igaki T: Mitochondrial defects trigger proliferation of neighbouring cells via a senescence-associated secretory phenotype in *Drosophila*. *Nat Commun.* 2014;5:5264. 10.1038/ncomms6264 25345385

[46] Freund A Laberge RM Demaria M: Lamin B1 loss is a senescence-associated biomarker. *Mol Biol Cell.* 2012;23(11):2066–75. 10.1091/mbc.E11-10-0884 22496421 PMC3364172

[47] Biteau B Karpac J Supoyo S: Lifespan extension by preserving proliferative homeostasis in *Drosophila*. *PLoS Genet.* 2010;6(10):e1001159. 10.1371/journal.pgen.1001159 20976250 PMC2954830

[48] López-Otín C Blasco MA Partridge L: The hallmarks of aging. *Cell.* 2013;153(6):1194–217. 10.1016/j.cell.2013.05.039 23746838 PMC3836174

[49] Green DR Galluzzi L Kroemer G: Mitochondria and the autophagy-inflammation-cell death axis in organismal aging. *Science.* 2011;333(6046):1109–12. 10.1126/science.1201940 21868666 PMC3405151

[50] Kriete A Mayo KL: Atypical pathways of NF-kappaB activation and aging. *Exp Gerontol.* 2009;44(4):250–5. 10.1016/j.exger.2008.12.005 19174186

[51] Rana A Oliveira MP Khamoui AV: Promoting Drp1-mediated mitochondrial fission in midlife prolongs healthy lifespan of *Drosophila melanogaster*. *Nat Commun.* 2017;8(1):448. 10.1038/s41467-017-00525-4 28878259 PMC5587646

[52] Odnokoz O Nakatsuka K Klichko VI: Mitochondrial peroxiredoxins are essential in regulating the relationship between *Drosophila* immunity and aging. *Biochim Biophys Acta.* 2017;1863(1):68–80. 10.1016/j.bbadis.2016.10.017 27770625 PMC5154953

[53] Parsons B Foley E: Cellular immune defenses of *Drosophila melanogaster*. *Dev Comp Immunol.* 2016;58:95–101. 10.1016/j.dci.2015.12.019 26748247

[54] Vlisidou I Wood W: *Drosophila* blood cells and their role in immune responses. *FEBS J.* 2015;282(8):1368–82. 10.1111/febs.13235 25688716

[55] Honti V Csordás G Kurucz É: The cell-mediated immunity of *Drosophila melanogaster*: hemocyte lineages, immune compartments, microanatomy and regulation. *Dev Comp Immunol.* 2014;42(1):47–56. 10.1016/j.dci.2013.06.005 23800719

[56] Haine ER Moret Y Siva-Jothy MT: Antimicrobial defense and persistent infection in insects. *Science.* 2008;322(5905):1257–9. 10.1126/science.1165265 19023083

[57] Guillou A Troha K Wang H: The *Drosophila* CD36 Homologue *croquemort* Is Required to Maintain Immune and Gut Homeostasis during Development and Aging. *PLoS Pathog.* 2016;12(10):e1005961. 10.1371/journal.ppat.1005961 27780230 PMC5079587

[58] Mackenzie DK Bussière LF Tinsley MC: Senescence of the cellular immune response in *Drosophila melanogaster*. *Exp Gerontol.* 2011;46(11):853–9. 10.1016/j.exger.2011.07.004 21798332

[59] Horn L Leips J Starz-Gaiano M: Phagocytic ability declines with age in adult *Drosophila* hemocytes. *Aging Cell.* 2014;13(4):719–28. 10.1111/acel.12227 24828474 PMC4116448

[60] Ramsden S Cheung YY Seroude L: Functional analysis of the *Drosophila* immune response during aging. *Aging Cell.* 2008;7(2):225–36. 10.1111/j.1474-9726.2008.00370.x 18221416

[61] Felix TM Hughes KA Stone EA: Age-specific variation in immune response in *Drosophila melanogaster* has a genetic basis. *Genetics.* 2012;191(3):989–1002. 10.1534/genetics.112.140640 22554890 PMC3389989

[62] Lesser KJ Paiusi IC Leips J: Naturally occurring genetic variation in the age-specific immune response of *Drosophila melanogaster*. *Aging Cell.* 2006;5(4):293–5. 10.1111/j.1474-9726.2006.00219.x 16803580

[63] Duneau D Ferdy JB Revah J: Stochastic variation in the initial phase of bacterial infection predicts the probability of survival in *D. melanogaster*. *eLife.* 2017;6: pii: e28298. 10.7554/eLife.28298 29022878 PMC5703640

[64] Kubiak M Tinsley MC: Sex-Specific Routes To Immune Senescence In *Drosophila melanogaster*. *Sci Rep.* 2017;7(1): 10417. 10.1038/s41598-017-11021-6 28874758 PMC5585412

[65] Sansone C Cohen J Gold B: Aging-associated dysbiosis increases susceptibility to enteric viral infection in *Drosophila* .2017 10.1101/156455

[66] Burger JM Hwangbo DS Corby-Harris V: The functional costs and benefits of dietary restriction in *Drosophila*. *Aging Cell.* 2007;6(1):63–71. 10.1111/j.1474-9726.2006.00261.x 17266676

[67] Shirasu-Hiza MM Schneider DS: Confronting physiology: how do infected flies die? *Cell Microbiol.* 2007;9(12):2775–83. 10.1111/j.1462-5822.2007.01042.x 17883424

[68] Schmid-Hempel P: Evolutionary ecology of insect immune defenses. *Annu Rev Entomol.* 2005;50:529–51. 10.1146/annurev.ento.50.071803.130420 15471530

[69] Sadd BM Siva-Jothy MT: Self-harm caused by an insect's innate immunity. *Proc Biol Sci.* 2006;273(1600):2571–4. 10.1098/rspb.2006.3574 16959651 PMC1634900

[70] Pursall ER Rolff J: Immune responses accelerate ageing: proof-of-principle in an insect model. *PLoS One.* 2011;6(5):e19972. 10.1371/journal.pone.0019972 21625631 PMC3097213

[71] Zhao P Lu Z Strand MR: Antiviral, anti-parasitic, and cytotoxic effects of 5,6-dihydroxyindole (DHI), a reactive compound generated by phenoloxidase during insect immune response. *Insect Biochem Mol Biol.* 2011;41(9):645–52. 10.1016/j.ibmb.2011.04.006 21554953 PMC3129360

[72] Koella JC Boëte C: A genetic correlation between age at pupation and melanization immune response of the yellow fever mosquito *Aedes aegypti*. *Evolution.* 2002;56(5):1074–9. 10.1111/j.0014-3820.2002.tb01419.x 12093022

[73] Ye YH Chenoweth SF McGraw EA: Effective but costly, evolved mechanisms of defense against a virulent opportunistic pathogen in *Drosophila melanogaster*. *PLoS Pathog.* 2009;5(4):e1000385. 10.1371/journal.ppat.1000385 19381251 PMC2663048

[74] Ma J Benson AK Kachman SD: *Drosophila melanogaster* Selection for Survival of *Bacillus cereus* Infection: Life History Trait Indirect Responses. *Int J Evol Biol.* 2012;2012:935970. 10.1155/2012/935970 23094195 PMC3474238

[75] Páez DJ Fleming-Davies AE Dwyer G: Effects of pathogen exposure on life-history variation in the gypsy moth ( *Lymantria dispar*). *J Evol Biol.* 2015;28(10):1828–39. 10.1111/jeb.12699 26201381 PMC4604033

[76] Fellowes Kraaijeveld Godfray: The relative fitness of *Drosophila melanogaster* (Diptera, Drosophilidae) that have successfully defended themselves against the parasitoid *Asobara tabida* (Hymenoptera, Braconidae). *J Evol Biol.* 1999;12(1):123–8. 10.1046/j.1420-9101.1999.00018.x

[77] Schwenke RA Lazzaro BP Wolfner MF: Reproduction-Immunity Trade-Offs in Insects. *Annu Rev Entomol.* 2016;61:239–56. 10.1146/annurev-ento-010715-023924 26667271 PMC5231921

[78] Flatt T Kawecki TJ: Juvenile hormone as a regulator of the trade-off between reproduction and life span in *Drosophila melanogaster*. *Evolution.* 2007;61(8):1980–91. 10.1111/j.1558-5646.2007.00151.x 17683439

[79] Jacot A Scheuber H Kurtz J: Juvenile immune system activation induces a costly upregulation of adult immunity in field crickets *Gryllus campestris*. *Proc Biol Sci.* 2005;272(1558):63–9. 10.1098/rspb.2004.2919 15875571 PMC1634936

[80] Moret Y Schmid-Hempel P: Survival for immunity: the price of immune system activation for bumblebee workers. *Science.* 2000;290(5494):1166–8. 10.1126/science.290.5494.1166 11073456

[81] Khan I Agashe D Rolff J: Early-life inflammation, immune response and ageing. *Proc Biol Sci.* 2017;284(1850): Pii: 20170125. 10.1098/rspb.2017.0125 28275145 PMC5360934

[82] Schwenke RA Lazzaro BP: Juvenile Hormone Suppresses Resistance to Infection in Mated Female *Drosophila melanogaster*. *Curr Biol.* 2017;27(4):596–601. 10.1016/j.cub.2017.01.004 28190728 PMC5319889

[83] Arrese EL Soulages JL: Insect fat body: energy, metabolism, and regulation. *Annu Rev Entomol.* 2010;55:207–25. 10.1146/annurev-ento-112408-085356 19725772 PMC3075550

[84] Chambers MC Song KH Schneider DS: *Listeria monocytogenes* infection causes metabolic shifts in *Drosophila melanogaster*. *PLoS One.* 2012;7(12):e50679. 10.1371/journal.pone.0050679 23272066 PMC3521769

[85] Dionne MS Pham LN Shirasu-Hiza M: *Akt* and *FOXO* dysregulation contribute to infection-induced wasting in *Drosophila*. *Curr Biol.* 2006;16(20):1977–85. 10.1016/j.cub.2006.08.052 17055976

[86] DiAngelo JR Bland ML Bambina S: The immune response attenuates growth and nutrient storage in *Drosophila* by reducing insulin signaling. *Proc Natl Acad Sci U S A.* 2009;106(49):20853–8. 10.1073/pnas.0906749106 19861550 PMC2791644

[87] Libert S Chao Y Zwiener J: Realized immune response is enhanced in long-lived *puc* and *chico* mutants but is unaffected by dietary restriction. *Mol Immunol.* 2008;45(3):810–7. 10.1016/j.molimm.2007.06.353 17681604

[88] Becker T Loch G Beyer M: FOXO-dependent regulation of innate immune homeostasis. *Nature.* 2010;463(7279):369–73. 10.1038/nature08698 20090753

[89] Rynes J Donohoe CD Frommolt P: Activating transcription factor 3 regulates immune and metabolic homeostasis. *Mol Cell Biol.* 2012;32(19):3949–62. 10.1128/MCB.00429-12 22851689 PMC3457521

[90] Clark RI Tan SW Péan CB: MEF2 is an *in vivo* immune-metabolic switch. *Cell.* 2013;155(2):435–47. 10.1016/j.cell.2013.09.007 24075010 PMC3807682

[91] Vrailas-Mortimer A del Rivero T Mukherjee S: A muscle-specific p38 MAPK/Mef2/MnSOD pathway regulates stress, motor function, and life span in *Drosophila*. *Dev Cell.* 2011;21(4):783–95. 10.1016/j.devcel.2011.09.002 22014527 PMC3199449

[92] Karpac J Biteau B Jasper H: Misregulation of an adaptive metabolic response contributes to the age-related disruption of lipid homeostasis in *Drosophila*. *Cell Rep.* 2013;4(6):1250–61. 10.1016/j.celrep.2013.08.004 24035390 PMC3832190

[93] Germain RN: The art of the probable: system control in the adaptive immune system. *Science.* 2001;293(5528):240–5. 10.1126/science.1062946 11452112

[94] Sarup P Sørensen P Loeschcke V: Flies selected for longevity retain a young gene expression profile. *Age (Dordr).* 2011;33(1):69–80. 10.1007/s11357-010-9162-8 20607427 PMC3063640

[95] Petersen AJ Katzenberger RJ Wassarman DA: The innate immune response transcription factor relish is necessary for neurodegeneration in a *Drosophila* model of ataxia-telangiectasia. *Genetics.* 2013;194(1):133–42. 10.1534/genetics.113.150854 23502677 PMC3632461

[96] Le Bourg E Malod K Massou I: The NF-κB-like factor DIF could explain some positive effects of a mild stress on longevity, behavioral aging, and resistance to strong stresses in *Drosophila melanogaster*. *Biogerontology.* 2012;13(4):445–55. 10.1007/s10522-012-9389-0 22791143

[97] Erkosar B Leulier F: Transient adult microbiota, gut homeostasis and longevity: novel insights from the *Drosophila* model. *FEBS Lett.* 2014;588(22):4250–7. 10.1016/j.febslet.2014.06.041 24983497

[98] Ryu JH Kim SH Lee HY: Innate immune homeostasis by the homeobox gene caudal and commensal-gut mutualism in *Drosophila*. *Science.* 2008;319(5864):777–82. 10.1126/science.1149357 18218863

[99] Fernando MD Kounatidis I Ligoxygakis P: Loss of Trabid, a new negative regulator of the *Drosophila* immune-deficiency pathway at the level of TAK1, reduces life span. *PLoS Genet.* 2014;10(2):e1004117. 10.1371/journal.pgen.1004117 24586180 PMC3930493

[100] Paredes JC Welchman DP Poidevin M: Negative regulation by amidase PGRPs shapes the *Drosophila* antibacterial response and protects the fly from innocuous infection. *Immunity.* 2011;35(5):770–9. 10.1016/j.immuni.2011.09.018 22118526

[101] Bonnay F Cohen-Berros E Hoffmann M: big bang gene modulates gut immune tolerance in *Drosophila*. *Proc Natl Acad Sci U S A.* 2013;110(8):2957–62. 10.1073/pnas.1221910110 23378635 PMC3581892

[102] Tan L Schedl P Song HJ: The *Toll*-->NFkappaB signaling pathway mediates the neuropathological effects of the human Alzheimer's Abeta42 polypeptide in *Drosophila*. *PLoS One.* 2008;3(12):e3966. 10.1371/journal.pone.0003966 19088848 PMC2597734

[103] Cao Y Chtarbanova S Petersen AJ: Dnr1 mutations cause neurodegeneration in *Drosophila* by activating the innate immune response in the brain. *Proc Natl Acad Sci U S A.* 2013;110(19):E1752–60. 10.1073/pnas.1306220110 23613578 PMC3651420

[104] Loch G Zinke I Mori T: Antimicrobial peptides extend lifespan in *Drosophila*. *PLoS One.* 2017;12(5):e0176689. 10.1371/journal.pone.0176689 28520752 PMC5435158

[105] Zhao HW Zhou D Haddad GG: Antimicrobial peptides increase tolerance to oxidant stress in *Drosophila melanogaster*. *J Biol Chem.* 2011;286(8):6211–8. 10.1074/jbc.M110.181206 21148307 PMC3057857

[106] Remolina SC Chang PL Leips J: Genomic basis of aging and life-history evolution in *Drosophila melanogaster*. *Evolution.* 2012;66(11):3390–403. 10.1111/j.1558-5646.2012.01710.x 23106705 PMC4539122

[107] Carnes MU Campbell T Huang W: The Genomic Basis of Postponed Senescence in *Drosophila melanogaster*. *PLoS One.* 2015;10(9):e0138569. 10.1371/journal.pone.0138569 26378456 PMC4574564

[108] Rose MR: Laboratory Evolution of Postponed Senescence in *Drosophila melanogaster*. *Evolution.* 1984;38(5):1004–10. 10.1111/j.1558-5646.1984.tb00370.x 28555803

[109] Ottaviani E Ventura N Mandrioli M: Gut microbiota as a candidate for lifespan extension: an ecological/evolutionary perspective targeted on living organisms as metaorganisms. *Biogerontology.* 2011;12(6):599–609. 10.1007/s10522-011-9352-5 21814818

[110] Broderick NA Lemaitre B: Gut-associated microbes of *Drosophila melanogaster*. *Gut Microbes.* 2012;3(4):307–21. 10.4161/gmic.19896 22572876 PMC3463489

[111] Bell G: Pathogen evolution within host individuals as a primary cause of senescence. *Genetica.* 1993;91(1–3):21–34. 10.1007/BF01435985 8125270 PMC7087926

[112] Ryu JH Ha EM Lee WJ: Innate immunity and gut-microbe mutualism in *Drosophila*. *Dev Comp Immunol.* 2010;34(4):369–76. 10.1016/j.dci.2009.11.010 19958789

[113] Schmid MR Anderl I Vesala L: Control of *Drosophila* blood cell activation via Toll signaling in the fat body. *PLoS One.* 2014;9(8):e102568. 10.1371/journal.pone.0102568 25102059 PMC4125153

[114] Eleftherianos I Castillo JC: Molecular mechanisms of aging and immune system regulation in *Drosophila*. *Int J Mol Sci.* 2012;13(8):9826–44. 10.3390/ijms13089826 22949833 PMC3431831

[115] Panayidou S Apidianakis Y: Regenerative inflammation: lessons from *Drosophila* intestinal epithelium in health and disease. *Pathogens.* 2013;2(2):209–31. 10.3390/pathogens2020209 25437036 PMC4235722

